# Supplementary material for: Long COVID in the United States
Source: PLoS One. 2023 Nov 2;18(11):e0292672. doi: 10.1371/journal.pone.0292672 (PMC10621843; doi:10.1371/journal.pone.0292672)
Supplement: S1 Appendix — (DOCX) [file pone.0292672.s001.docx]

**Appendix 1. Questions used to identify Long COVID – with weighted percentages in square parentheses.**

Q1. Did you have any symptoms lasting 3 months or longer that you did not have prior to having coronavirus or COVID-19? Long term symptoms may include - tiredness or fatigue, difficulty thinking, concentrating, forgetfulness, or memory problems (sometimes referred to as "brain fog", difficulty breathing or shortness of breath, joint or muscle pain, fast-beating or pounding heart (also known as heart palpitations), chest pain, dizziness on standing, menstrual changes, changes to taste/smell, or inability to exercise? [14.4%]

Q2. Have you ever tested (using a rapid point-of-care test, self-test, or laboratory test) positive for COVID-19 or been told by a doctor or other health care provider that you have or had COVID-19? [46.7%]

Q3. Have you ever had COVID-19 or coronavirus symptoms lasting 3 months or longer?[14.4%]

Q4. Do you currently have COVID-19 or coronavirus symptoms? [8.6%]

Q5. How would you describe the COVID-19 symptoms when they were at their worst – (of those who had COVID);

i) I had no symptoms [6.4%]

ii) I had mild symptoms [39.9%]

iii) I had moderate symptoms [40.4%]

iv) I had severe symptoms [13.3%].

Q6. Do these long-term symptoms reduce your ability to carry out day-to-day activities compared with the time before you had COVID-19?

0) No long COVID [93.7%]

1) Yes, a lot [1.4%]

2) Yes, a little [3.6%]

3) Not at all [1.3%]

Q7. Over the last 2 weeks, how often have you been bothered by feeling nervous, anxious, or on edge? Select only one answer.

1) Not at all [39.0%]

2) Several days [33.0%]

3) More than half the days [11.8%]

4) Nearly every day [16.3%]

Q8. Over the last 2 weeks, how often have you been bothered by the not being able to stop or control worrying? Select only one answer.

1) Not at all [46.1%]

2) Several days [30.9%]

3) More than half the days [10.1%]

4) Nearly every day [12.9%]

Q9. Over the last 2 weeks, how often have you been bothered by feeling down, depressed, or hopeless? Select only one answer.

1) Not at all [51.5%]

2) Several days [29.3%]

3) More than half the days [8.8%]

4) Nearly every day [10.4%]

Q10. Over the last 2 weeks, how often have you been bothered by having little interest or pleasure in doing things? Select only one answer.

1) Not at all [50.2%]

2) Several days [29.1%]

3) More than half the days [10.4%]

4) Nearly every day [10.3%]

Q11. Do you have difficulty walking or climbing stairs? Select one.

1) No - no difficulty [73.0%]

2) Yes - some difficulty [20.7%]

3) Yes - a lot of difficulty [5.6%]

4) Cannot do at all [0.7%]

Q12. Do you have difficulty with self-care, such as washing all over or dressing? Select one.

1) No - no difficulty [91.1%]

2) Yes - some difficulty [7.4%]

3) Yes - a lot of difficulty [1.2%]

4) Cannot do at all [0.4%]

Q13. Using your usual language, do you have difficulty communicating, for example understanding or being understood? Select one.

1) No - no difficulty [91.2%]

2) Yes - some difficulty [7.5%]

3) Yes - a lot of difficulty [1.0%]

4) Cannot do at all [0.3%]

Q14. Do you have difficulty remembering or concentrating? Select one.

1) No - no difficulty [55.5%]

2) Yes - some difficulty [37.2%]

3) Yes - a lot of difficulty [6.9%]

4) Cannot do at all [0.4%]
