# Supplementary material for: Long COVID in the United States
Source: PLoS One. 2023 Nov 2;18(11):e0292672. doi: 10.1371/journal.pone.0292672 (PMC10621843; doi:10.1371/journal.pone.0292672)
Supplement: Attachment — Submitted filename: PLOS Jul23.docx [file pone.0292672.s002.docx]

| **PDF Page, Line** | **Comment** |
| --- | --- |
| 43 - Abstract | It “peaks in midlife” -Can we refer to long covid as peaking given the relatively short duration of this event, not allowing for an appropriate follow-up period? |
| 45, 28 | The paragraph in the Introduction is a repeat of the Abstract. What’s the purpose? Here again is the phrase about peaking in midlife. |
| 45, 33-34 | “The effect is larger …” What is being referred to here, is it long COVID (LC)? And which groups are being compared? Is it the effect of LC is larger among people who currently report LC compared to those who experienced LC in the past? |
| 46, 48-50 | Italics and quotation marks are not used in a consistent way. |
| 46, 55 | The authors’ recap of O’Mahoney et al’s findings – is ‘COVID-19 survivors’ the same as anyone who had COVID? |
| 46, 62-63 | Results of Taquet are confusing – was a larger proportion of survivors (57%) affected after 180 days than in the earlier period (37% in 90 to 180 days)? |
| 46, 65 > (paragraph) | Is there a reason for this paragraph to be written in the present tense? |
| 47, 79 >  47, 84 | Rather than per population, could we report on the per cent of the survivors with long COVID?  ‘They adversely impacted …’ is ‘they’ referring to long COVID? The earlier use of ‘they’ in that paragraph referred to the Office of National Statistics. |
| 52, 185 till Wed |  |
| 59, 345 | Is this a table of results from the current study? The content focuses on anxiety. However, I do not recall details about the measure of anxiety and how the scores are interpreted in the Methods section. 58, 318 – seems to be referring to mental health data and coding, which should be expanded in the Methods. |
| 59, 342 | Is the 15% referring to the 14% in the data presented on anxiety? … in the ‘non COVID’ group. |
| 60, 374 | “The four long COVID categories each have positive signs..” unclear what this means. |
|  | Table 5 Notes – it is unclear what is meant by ‘age dummies’ and in subsequent tables ‘Working/cut1/ cut2 etc. These are not explained or described. |
|  | Table 6 – conventional formatting of this and other tables might help the reader to understand what is being presented. |
|  | Chart 1 differ from Figure 1? |
| 61, 389 | Isn’t the reference to Wang et al discussing what this paper defines as ‘long COVID’? What is ‘post-COVID-19 conditions” and is the ‘?’ a typo? |
| 61-62 | The authors’ interpretation of the findings is not provided; there is no Discussion. The Conclusions section provides a summary of the findings. |
